# Perceptual learning with dichoptic attention tasks improves attentional modulation in V1 and IPS and reduces interocular suppression in human amblyopia

**DOI:** 10.1038/s41598-022-13747-4

**Published:** 2022-06-11

**Authors:** Chuan Hou, Spero C. Nicholas

**Affiliations:** The Smith-Kettlewell Eye Research Institute, San Francisco, CA 94115 USA

**Keywords:** Neuroscience, Cognitive neuroscience, Visual system, Psychology

## Abstract

Long-term and chronic visual suppression to the non-preferred eye in early childhood is a key factor in developing amblyopia, as well as a critical barrier to treat amblyopia. To explore the relationship between selective visual attention and amblyopic suppression and its role in the success of amblyopic training, we used EEG source-imaging to show that training human adults with strabismic and anisometropic amblyopia with dichoptic attention tasks improved attentional modulation of neural populations in the primary visual cortex (V1) and intraparietal sulcus (IPS). We also used psychophysics to show that training reduced interocular suppression along with visual acuity and stereoacuity improvements. Importantly, our results revealed that the reduction of interocular suppression by training was significantly correlated with the improvement of selective visual attention in both training-related and -unrelated tasks in the amblyopic eye, relative to the fellow eye. These findings suggest a relation between interocular suppression and selective visual attention bias between eyes in amblyopic vision, and that dichoptic training with high-attention demand tasks in the amblyopic eye might be an effective way to treat amblyopia.

## Introduction

The human brain suppresses visual input from the non-fixating eye to prevent double vision (diplopia) when the eyes become misaligned (strabismus)^1–5^, or suppresses the blurred image from the eye with higher refractive error in anisometropia^4–6^. Long-term and chronic visual suppression to the non-preferred eye is a key factor causing amblyopia (i.e., reduced visual acuity in the non-preferred eye)^4^, as well as a critical barrier to treat amblyopia^7–9^. Amblyopia affects about 3–5% of the population^10^ and is the leading cause of monocular vision loss in children^11^. The standard clinical treatment consists of best optical correction and patching the fellow eye (occlusion therapy) in children and teenagers. However, recent research using perceptual learning has also shown improvements in various visual functions in adults with amblyopia^12–16^.

Using diverse methodologies, almost all studies with behavioral training have shown improvements in visual acuity and other visual functions in amblyopia over the past decades^14^. Goal-driven tasks (completing specific tasks to achieve a planned outcome) and behavioral training (e.g., perceptual learning) commonly involve top-down attentional control^17,18^, reweight the responses of neurons activated by the trained stimulus^19^ and engage high-level cognitive compensation^20^, which may underlie the neural basis of improving visual function. For example, using dichoptic video games^21–26^ or monocular action video games^27^ in adults and children with amblyopia result in a marked improvement of visual acuity and/or stereoacuity. Li et al.^27,28^ reported that attention capacity also improved after monocular videogame play in adults with amblyopia. These studies imply that the engagement of attention in the amblyopic eye in videogame play might be an important factor for successful training. Indeed, there is compelling evidence in the literature that action game play enhances selective visual attention in space (more effective localization of a target among distractors^29^) and time (enhancing the ability to select relevant information over time^30^). However, the role of attention in the success of amblyopic training has not been systematically studied.

Another important factor for the success of amblyopic training might be the dichoptic approach that engages binocular function with “balanced contrast”^21–24,26,31^, where the physical contrast in the amblyopic eye is increased relative to the fellow eye to equate perceptual visibility, also known as “anti-suppression therapy” proposed by Hess et al.^31^. Learning with this strategy^22^ or combining this strategy with visual cueing to the amblyopic eye (push–pull treatment)^32^ resulted in a marked reduction of interocular suppression along with visual acuity/stereoacuity improvement in adults with amblyopia. We speculate that increasing contrast to the stimuli of the amblyopic eye might strengthen excitatory interactions through a bottom-up visual salience and attention process^33^. Supporting this point of view, our recent study in humans^34^ and another study in monkeys^35^ have found that excitatory contribution to binocular interactions in the visual cortex is preferentially reduced in strabismic amblyopia. Strengthening bottom-up excitatory connections may help restore latent binocular capacity, thus alleviating suppression. A number of studies that used repetitive transcranial magnetic stimulation (rTMS) that improved contrast sensitivity^36,37^ and stereoacuity^38^ in adults with amblyopia also support this point of view. According to the framework of attention, the bottom-up attention process can be strongly modulated or even overridden by top-down, user-driven factors (i.e., goal-driven tasks)^39–41^. Therefore, amblyopic training with a balanced contrast strategy, which has been demonstrated as a successful anti-suppression therapy^22,32^, may engage both bottom-up and top-down attentional processes. However, what neural basis underlies this successful anti-suppression therapy, and what role, if any, attention plays in visual suppression of amblyopic vision are still open questions.

**Figure 1:**
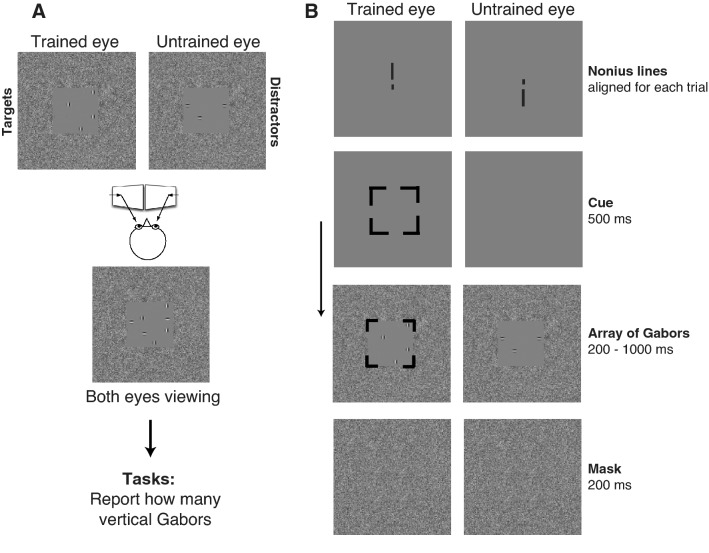
Illustration of perceptual learning stimuli. (**A**) Perception of a given trial with targets (vertical Gabors) in the trained eye and distractors (horizontal Gabors) in the untrained eye under dichoptic viewing through a mirror stereoscope. (**B**) The temporal sequence of a given trial in perceptual learning sessions. A random array of highly visible Gabor patches was presented in the trained eye with vertical Gabors (targets) and in the untrained eye with horizontal Gabors (distractors) followed by a 200 ms noise mask. A 500 ms-valid attentive cue preceded each trial indicating which eye would get the targets. The trials were self-initiated and participants were requested to respond as accurately as possible with no time limit and no feedback was given.

We previously reported that the magnitude of interocular suppression correlates with degraded attentional modulation of neurons in the primary visual cortex (V1) to visual input from the amblyopic eye in adults with strabismic amblyopia^42^. To further explore the precise relationship between selective visual attention and amblyopic suppression and its role in the success of amblyopic training, in the present study, we aimed to test whether perceptual learning with dichoptic attention tasks (1) improves attentional modulation in visual cortex and (2) reduces interocular suppression in adults with amblyopia, and (3) whether there is a correlation between the improvement of attentional modulation in visual cortex and the reduction of interocular suppression. We designed perceptual learning tasks that avoided typical contrast sensitivity-based tasks and required a significant degree of attentional effort for the trained eye with a dichoptic approach, as illustrated in Fig. 1. Our training design included three key factors (searching^43–45^, counting^46^, and attentional cueing^47,48^) implemented selective visual attention in the tasks, and thus incorporated “high attention-demand”. The dichoptic attention tasks consisted of quickly *searching* and *counting* “targets” presented to the trained eye among “distractors” that were simultaneously presented to the untrained eye preceded by *attentional cueing* to the trained eye. We provided a 100% valid cue to the trained eye, because our own pilot study in human amblyopes^49^ and other studies in human^50,51^ and monkey^52^ amblyopes have shown that cueing the amblyopic eye improves task performance. Noted that it is not clear whether and how much the cue contributed to attentional selection because we did not have an invalid cue. Therefore, although a cue was included in the study, we cannot quantify its effect on the attentional task, even if the participants might have used the cue. We arranged targets with 90% of the trials in the amblyopic eye and with only 10% of the trials in the fellow eye in a random order within a block. We expected to improve attention deployment considerably in the amblyopic eye, and thus to reduce attention allocation bias to the fellow eye that is typically found in adults with strabismic amblyopia^42,49^. We trained adults with both strabismic and anisometropic amblyopia. Training sessions were 2 h each with about two visits per week for two months. Attentional modulation of neural populations in V1 and intraparietal sulcus (IPS) and interocular suppression along with visual acuity and stereoacuity were measured and compared before and after the training. We also tracked the change of attention allocation bias between the eyes and the magnitude of interocular suppression as well as visual acuity across perceptual learning sessions in some participants.

## Results

### Perceptual learning improved selective attention with trained task

**Figure 2:**
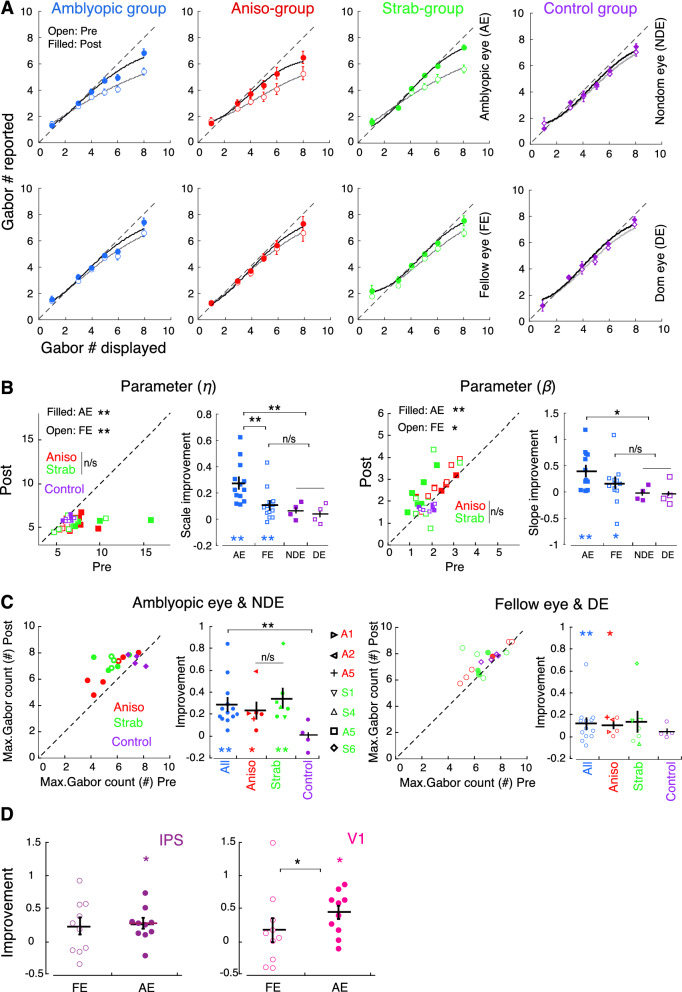
Selective visual attention with trained and untrained tasks pre- and post-perceptual learning (PL). (**A**) Group mean of attention improvement with trained tasks (searching/counting performance). Colors denote the group. Error bars denote SEM. Dashed lines indicate 1:1 ratio between reported and displayed number of Gabors. Solid curves are the fits of a variant of the Weibull function. (**B**) Comparison of Weibull function fit results from individual participants in trained tasks pre- and post-PL with the fit parameters *η* (left 2 panels) and *β* (right 2 panels). Data below the dashed line (1:1 ratio of pre- and post-PL) for parameter *η* and above the dashed line for parameter *β* indicate improvement after training. (**C**) Comparison of Max. Gabor count from individual participants pre- and post-PL. Data above the dashed lines indicate improved performance in the trained tasks. Four participants who did not complete 16 PL sessions are marked with open symbols for the AE in the left panel and with filled symbols for the FE in the third panel in (**B**) and (**C**). Participants who were tested repeatedly across PL sessions in “Results” section “Correlation of task-related selective attention and interocular suppression/visual acuity across perceptual learning sessions” were identified by different symbols in the second and fourth panels of (**C**). (**D**) Individual data for the improvement of attentional modulation in neuronal populations of IPS and V1 with the untrained tasks measured by source-imaged SSVEP. Error bars denote SEM. *, and ** denote *p* < 0.05 and *p* < 0.01, respectively. Black asterisks indicate paired tests and colored asterisks indicate one-sample tests. Statistical analysis included all participants. Extended data for (**A**) and (**C**) with searching and counting errors are shown in Fig. S1.

Visual counting, particularly when counting large set-sizes of Gabors (i.e., Gabor set-sizes beyond 5)^46^, requires rapid shifts in attention and involves visual attention processes^50,51^, as well as engages high-level visual pathways^52^ and the intraparietal sulcus (IPS)^53,54^, a region known to be involved in visual attention^55^. Our previous work^56,57^ and others^58^ have shown that counting with large set-sizes of Gabors is impaired in the amblyopic eye, and that it is further impaired under conditions of interocular suppression^56^. Thus, we used feature counting in perceptual learning and expected to improve attention deployment to visual input from the amblyopic eye, which allowed us to compare attentional modulation of neural populations in IPS^53,54^ as well as in V1^42^ before and after the training. Like any perceptual learning (PL), searching and counting performance improved in both the amblyopic eye (AE) and the fellow eye (FE) of amblyopes after repetitive practice of the tasks. This is evident in Fig. 2A where the reported number of Gabors was closer to the displayed number of Gabors after perceptual learning, compared to that before perceptual learning. The data plotted in Fig. 2 were the measurements before and after training with 35% contrast in each eye at 200 ms stimulus duration across all participants (amblyopes, n = 13; normal-vision controls, n = 4). As our training stimuli incorporated high attention-demand tasks, thus, the task-performance improvement was also considered to represent attention deployment improvement in the trained eye. Underestimating visual features is one of the characteristics of amblyopic deficits, which is believed to occur due to dysfunction of high-level cortex and spatial attention^56–58^. This feature-underestimating phenomenon was also observed in our study (Fig. 2A, top row, open symbols in left three panels). However, this underestimation was reduced after perceptual learning (Fig. 2A, top row, filled symbols in left three panels). Normal-vision controls also showed a similar improvement in counting as the fellow eye of amblyopes, in which the improvement was much less than the improvement in the amblyopic eye.

We quantified searching/counting performance by a variant of the Weibull cumulative distribution-function. The solid curves in Fig. 2A are the fits of Weibull function to the data of group mean. The results of fits in data of individual participants are shown in Fig. 2B, in which the average goodness of fit (R^2^) was 0.98 ± 0.02 (SD) across participants. In the very left panel of Fig. 2B, the fit parameter eta (*η*) was significantly smaller post-PL than pre-PL in both eyes of amblyopes (*df* = 12; AE: *t* = 4.0994, *p* = 0.0015; FE: *t* = 3.9664, *p* = 0.0019), indicating a leftward shift of Weibull function, indicating that the reported number of Gabors is closer to the displayed number of Gabors after perceptual learning. As seen in Fig. 2B (the second panel), the performance improvement was greater in the AE with an improvement of 27% on average compared to the improvement in the FE, which was 12% on average (*df* = 12, *t* = 3.8369, *p* = 0.0024) and in both eyes of controls (*df* = 16, *t* = 3.6369, *p* = 0.0066). No significant difference was found between Ansio and Strab subgroups (*df* = 12, AE: *t* = 0.5133, *p* = 0.6171; FE: *t* = 0.8892, *p* = 0.3914), which is consistent with the findings of attention deployment improvement after monocular video-game play training in adults with amblyopia^27^. No significant difference was found between the FE and both eyes of controls (*df* = 16, *t* = 0.5133, *p* = 0.365). The improvements in both eyes of amblyopes were also significant when compared to the pre-test baseline (*df* = 12, AE: *t* = 4.0994, *p* = 0.0015; FE: *t* = 3.9664, *p* = 0.0019). The improvements in both eyes of controls did not reach significance (p > 0.05) when compared to the pre-test baseline. The greater improvement in the AE than in the FE might be due to: (1) the latent attentional capability in the AE was revealed by PL with high attention-demand tasks, which had more room to improve compared to that in the FE; and (2) more repetitive practice of attention tasks in the AE (90% trials with targets) in each visit session compared to that in the FE with 10% trials with targets. In the third panel of Fig. 2B, the fit parameter beta (*β*), which measures how fast the function rises, was significantly larger post-PL than pre-PL in both eyes (*df* = 12; AE: *t* = 4.0994, *p* = 0.0015; FE: *t* = 2.2205, *p* = 0.0464), indicating that the reported number of Gabors is closer to the displayed number of Gabors after perceptual learning as well. The improvements in both eyes of controls did not reach significance (p > 0.05) when compared to the pre-test baseline. The improvement in the AE was significantly larger than both eyes of controls (*df* = 16, *t* = 2.583, *p* = 0.0157), but no significant difference was found between the FE and both eyes of controls (*df* = 16, *t* = 0.865, *p* = 0.2575) and between the AE and the FE (*df* = 12, *t* = 0.865, *p* = 0.2575).

Furthermore, we also compared the task-performance pre- and post-PL at a given maximum number of Gabor-set (referred to as Max. Gabor count) (Fig. 2C). The performance (Max. Gabor Count) of both the AE (the left panel in Fig. 2C; *df* = 12, *t* = 4.0994, *p* = 0.0015) and the FE (the third panel in Fig. 2C; *t* = 3.1249, *p* = 0.0088) significantly improved after perceptual learning, but not the both eyes of controls (*p* > 0.05). The AE had a greater improvement in comparison to the FE (*df* = 12, *t* = 3.8369, *p* = 0.0024) and to the both eyes of controls (*df* = 16, *t* = 2.921, *p* = 0.0046). The improvement was 29.6% in the AE (Fig. 2C, the second panel) and 12.5% in the FE (Fig. 2C, the right panel), and both were significant when compared to the baseline of no improvement (AE: *t* = 4.0994, *p* = 0.0015; FE: *t* = 2.2205, *p* = 0.0464), but the improvement in both eyes of controls was not significant when compared to the baseline of no improvement (*p* > 0.05). No significant difference was found between Aniso and Strab subgroups (*df* = 12, AE: *t* = 1.5252, *p* = 0.1531, FE: *t* = 0.8892, *p* = 0.3914). Thus, selective attention improvements with trained tasks (searching/counting performance) quantified by Weibull function (Fig. 2B) and Max. Gabor count (Fig. 2C) were consistent.

Note that there were four participants (marked with a different symbol in Fig. 2C) who did not complete all 16 PL sessions (see details in “Methods”: Perceptual learning strategy and procedure), but still showed improvement in performance. This might be because these four participants completed the fast-learning phase of perceptual learning (> 2600 trials), as fast-learning usually happens within the first 2–4 sessions (roughly 1000–2000 trials)^59–61^. Data from the last visit session for these four participants were used for the post-PL data in the current study. Statistical analysis included all participants. We also looked at searching and counting errors (defined as the difference between displayed and reported number of Gabors), which decreased after perceptual learning as well (data are shown in Supplementary Information, Fig. S1).

### Perceptual learning improved selective attention with untrained task

To determine whether the improvement of task-performance by dichoptic attention training accompanies the improvement of attention deployment, we used a separate attention task that was unrelated to the training task to evaluate the improvement of attention deployment in participants with amblyopia. We measured and compared attentional modulation in neuronal populations of IPS and V1 using source-imaged SSVEP before and after training for 10 out of 13 participants. Three participants, Participants A2, S6 and S7* did not complete the SSVEP measurement post-training. We focused on V1 because our earlier work using the same attention task showed degraded attentional modulation in V1 from visual input of the amblyopic eye^42^. We also focused on IPS, because this area is known to be involved in visual counting^53,54^. We found that attentional modulation in areas V1 and IPS in response to input from the amblyopic eye significantly improved when compared to the pre-perceptual learning baseline (IPS, in the left panel of Fig. 2D: *df* = 9, *t* = 2.262, *p* = 0.0297; V1, in the right panel of Fig. 2D: *df* = 9, *t* = 3.250, *p* = 0.0083), indicating attention deployment improvement in the amblyopic eye after dichoptic attention training. Attentional modulation improvement found in IPS area in the current study is consistent with previous studies that showed activation in IPS with counting tasks^53,54^. It is worth noting that attentional modulation improved to visual input from the fellow eye for some participants; however, these improvements did not reach significance in a group mean (V1: *df* = 9, *t* = 1.100, *p* = 0.2573; IPS: *df* = 9, *t* = 1.383, *p* = 0.1206). These might be due to a ceiling effect for the fellow eye, or due to less practice since only 10% of the trials with targets were presented to the fellow eye. The attentional modulation improvement was significantly greater for the amblyopic eye than for the fellow eye in V1 (*df* = 9, *t* = 2.262, *p* = 0.0463), but not in IPS (*df* = 9, *t* = 0.703, *p* = 0.3994). The similar improvement in IPS neurons to inputs from the two eyes might be because neurons in higher-level cortex typically combine signals from the two eyes.

### The reduction of interocular suppression after perceptual learning is correlated with the improvement of selective visual attention in both training-related and -unrelated tasks

**Figure 3:**
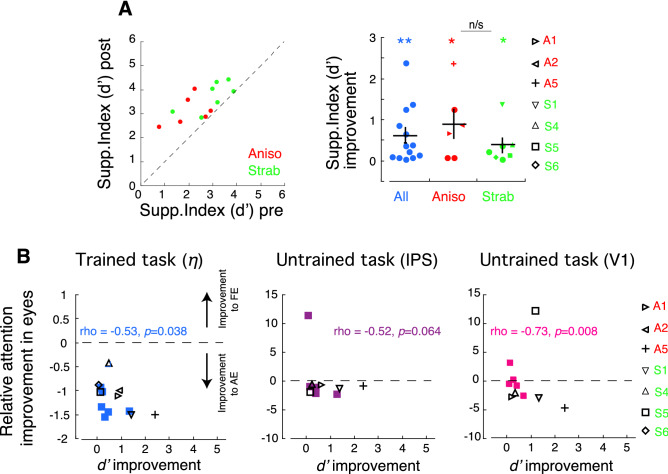
Relation between the improvements of selective visual attention and Supp. Index. (**A**) Supp. Index (*d*′) pre- and post-perceptual learning (PL). Data above the dashed line (1:1 ratio of Supp. Index pre- and post-PL) indicate improvement of Supp. Index. Error bars denote SEM. (**B**) Relation between the relative attention improvement in the eyes in selective attention with trained task (left panel) and with untrained task in IPS (middle panel) and in V1 (right panel) and improvement of Suppp. Index (*d*′). The horizontal dashed lines indicate that the improvement fraction is equal between the eyes. Data below the dashed lines indicate selective attention improved more in the amblyopic eye relative to the fellow eye, and vice versa. In middle (IPS) and right (V1) panels, the values of *rho* and *p* included an outlier. Without outlier, *rho* = − 0.33 (*p* = 0.1903) in IPS and *rho* = − 0.72 (*p* = 0.0149) in V1. *, and ** denote *p* < 0.05 and *p* < 0.01, respectively. The colored asterisks indicate one- sample tests. Participants who were tested repeatedly across PL sessions in “Results” section “Correlation of task-related selective attention and interocular suppression/visual acuity across perceptual learning sessions” were identified by different symbols.

We measured and compared interocular suppression represented by a Suppression Index (*d*′, referred to as Supp. Index; see detail in “Methods” and Supplementary Information) before and after perceptual learning (Fig. 3A) in participants with amblyopia, where larger *d*′ indicates less suppression. We did not include normal-vision controls in this experiment, because they had no visual suppression with this measure. The mean improvement in Supp. Index relative to the pre-training baseline was significant in the Aniso group (*df* = 5, *t* = 3.0723, *p* = 0.0139), the Strab group (*df* = 6, *t* = 2.8896, *p* = 0.0139) and the two subgroups combined (*df* = 12, *t* = 4.0994, *p* = 0.0007). The results indicate that *d*′ (Supp. Index) increased after training, reflecting that perceptual learning alleviated interocular suppression. These findings are, in general, consistent with previous reports of the reduction of interocular suppression using balanced-contrast training strategy^22,26,32,62^. Knox et al.^26^ reported that training reduced interocular suppression for only 7 out of 14 amblyopic children. This might be due to fewer training hours (only 5 h training), compared to other studies and ours (32 h in completed training). No significant difference was found in the improvement of Supp. Index between subgroups of Anisos and Strabs (*df* = 12, *t* = 0.8892, *p* = 0.3914). It is not clear whether there is a difference in reduction of interocular suppression between the Aniso and Strab groups using contrast-balanced strategy in previous studies with Tetris training in adults^22^ and children^26^ and with iPad training in children^21,24,63^, as these studies did not specifically compare interocular suppression between the subgroups (in Tetris training) or did not report interocular suppression (in iPad training).

Previous studies^42,49^ have shown that attention allocation is typically biased to the fellow eye in strabismic amblyopes. With our stimulus arrangement in 90% of the trials with high-attention demand tasks in the amblyopic eye and only 10% of the trials with attention tasks in the fellow eye in perceptual learning, we expected to induce a greater improvement in selective attention to the amblyopic eye than to the fellow eye. To show whether training reduces the habitual attention allocation bias to the fellow eye, we calculated reduction fraction of attention allocation bias to the fellow eye as [(FE post-PL − AE post-PL) − (FE pre-PL − AE pre-PL)]/(FE pre-PL − AE pre-PL), which basically reflects “*relative attention improvement in the eyes*”. As seen in Fig. 3B, although selective attention improved in both eyes after the training, the improvement was larger for the amblyopic eye relative to the fellow eye in both the trained task (Fig. 3B left panel, the fit parameter *η*) and the untrained task (Fig. 3B right panel, attentional modulation in V1), in which the majority of participants showed more improvement toward the amblyopic eye (points below the dashed lines). Interestingly, the improvement of Supp. Index did not correlate with the improvement of selective attention in the amblyopic eye *itself* (fit parameter *η*: *rho* = − 0.34, *p* = 0.1315; IPS: *rho* = 0.07, *p* = 0.4274; V1: *rho* = 0.18, *p* = 0.3136), but correlated with the *fraction of reduction in attention allocation bias to the fellow eye* when taking into account the *relative* attention improvements in the eyes for both trained tasks (Fig. 3B, left panel) and untrained tasks in V1 (Fig. 3B, left panel). However, this correlation was not found in IPS (Fig. 3B, middle panel). The striking finding in the present study was that the negative correlation of Supp. Index (*d*′) and selective attention were consistent in the measurements of the trained task (Fig. 3B, left panel) and the untrained task (Fig. 3B, right panel, attentional modulation in V1). This correlation indicated the more selective attention improvement to the amblyopic eye, the more reduction of interocular suppression (larger *d*′ of Supp. Index), suggesting a relation between interocular suppression and attention allocation bias between the eyes in amblyopic vision.

### Visual acuity and stereoacuity improved after perceptual learning

**Figure 4:**
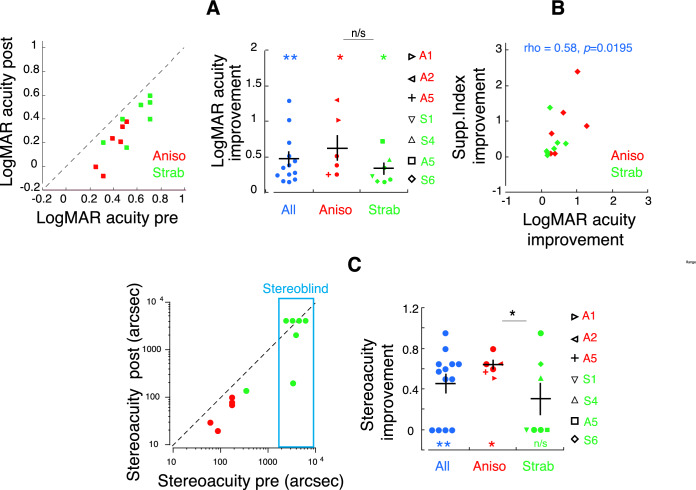
Visual function improvement after perceptual learning (PL). (**A**) LogMAR acuity pre- and post-PL. Data below the dashed line (1:1 ratio of logMAR acuity pre- and post-PL) indicate logMAR improvement. (**B**) Correlation between improvements of logMAR and Supp. Index (*d*′). (**C**) Stereoacuity pre- and post-PL. Data below the dashed line (1:1 ratio of stereoacuity pre- and post-PL) indicate stereoacuity improvement. Colors denote the group. Error bars denote SEM. Note that in (**C**), data with non-measurable stereoacuity were plotted as 4000 arcsec, shown in blue box. *, and ** denote p < 0.05 and p < 0.01, respectively. Black asterisks indicate paired tests and colored asterisks indicate one- sample tests. Participants who were tested repeatedly across PL sessions in “Results” section “Correlation of task-related selective attention and interocular suppression/visual acuity across perceptual learning sessions” were identified by different symbols in the right panels of (**A**) and (**C**).

LogMAR acuity improved with perceptual learning (Fig. 4A) and was significantly correlated with the improvement of Supp. Index (Fig. 4B). This finding is consistent with previous reports of a close relation between interocular suppression and visual acuity in amblyopia^2–4,42,64^. The group mean of logMAR acuity improvement (right panel in Fig. 4A) was significant when compared to the baseline in the Aniso group (*df* = 5, *t* = 3.3650, *p* = 0.0139), the Strab group (*df* = 6, *t* = 3.7070, *p* = 0.0089) and the two subgroups combined (*df* = 12, *t* = 4.3180, *p* = 0.0007). No significant difference was found between subgroups of Anisos and Strabs (*df* = 12, *t* = 1.7820, *p* = 0.0865), which is consistent with a previous study that showed no clear difference in visual acuity improvement between these subgroups^59^ (also see^12^ for review).

Stereoacuity also improved in 9 out 13 participants with perceptual learning, as shown in Fig. 4C. The group mean of improvement fraction in stereoacuity was significant when compared to the baseline in the Aniso group (*df* = 5, *t* = 2.571, *p* = 0.0139) and the two subgroups combined (*df* = 12, *t* = 4.318, *p* = 0.0038), but not in the Strab group (*df* = 6, *t* = 1.943, *p* = 0.0544). The improvement calculation for the participants who had non-measurable stereoacuity was assumed to be 4000 arcsec, for simplicity of quantification. The stereoacuity improvement was significantly larger in the Aniso group than in the Strab group (*df* = 12, *t* = 2.179, *p* = 0.0306).

More detailed description of improvements in visual acuity and stereoacuity with perceptual learning can be found in Supplementary Information.

### Comparison of training outcomes between equal contrast and balanced contrast strategies

**Figure 5:**
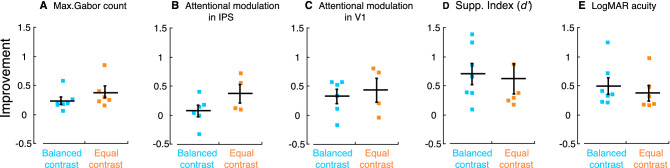
Comparison of the training outcomes in individual participants between balanced and equal contrasts in the eyes in perceptual learning. Improvement fractions in the amblyopic eye by perceptual learning from each participant were plotted for Max. Gabor count improvement (**A**), attentional modulation improvement in IPS (**B**) and in V1 (**C**), Supp. Index improvement (**D**) and logMAR acuity improvement (**E**). Colors denote the training strategies. Error bars denote SEM.

The comparison of the training outcomes from individual participants between the groups with two training strategies is shown in Fig. 5, where the data were re-plotted by regrouping participants from Figs. 2, 3 and 4. No significant differences in group mean were found in the training outcomes between balanced and equal contrast strategies in task-related selective attention improvement represented by Max. Gabor count (Fig. 5A; *df* = 12, *t* = 1.083, *p* = 0.1985), task-unrelated selective attention improvement represented by attentional modulation in IPS (Fig. 5B; *df* = 9, *t* = 1.833, *p* = 0.0550) and in V1 (Fig. 5C; *df* = 9, *t* = 0.000, *p* = 0.5224), reduction of interocular suppression represented by Supp. Index (Fig. 5D; *df* = 12, *t* = 0.695, *p* = 0.3914) and logMAR acuity improvement (Fig. 5E; *df* = 12, *t* = 0.873, *p* = 0.2531). Note that these statistical results were based on small sample sizes, although non-parametric tests were used. Thus, a direct visualization of the training outcomes from individual participants in Fig. 5 is preferable. We also tested pre-training visual acuity in the two training groups, and found no significant difference in logMAR acuity pre-training between the two groups (mean ± SD: 0.51 ± 0.06 in the balanced-contrast group; 0.47 ± 0.07 in the equal-contrast group; *df* = 12, *t* = 0.000, *p* = 0.6682).

### Correlation of task-related selective attention and interocular suppression/visual acuity across perceptual learning sessions

To investigate the relation between selective attention and visual suppression in amblyopia, presumably, the best way is to look at the change of interocular suppression over the time of perceptual learning with dichoptic attention tasks within subjects. We were able to measure and track interocular suppression in 7 out of 13 participants across multiple perceptual learning sessions. This allowed us to conduct correlation analysis across perceptual learning sessions within-subjects to further confirm whether improvement of selective attention in the amblyopic eye accompanies the reduction of visual suppression in amblyopes.

#### Correlation with perceptual learning sessions

The relation between Max. Gabor count, Supp. Index and logMAR acuity in the amblyopic eye with perceptual learning sessions in individual participants are shown in Supplementary Information (Fig. S2). The data showed that task-related selective attention and logMAR acuity progressively improve with perceptual learning sessions while interocular suppression decreases. Furthermore, we also observed a pattern of initial rapid improvement (fast-learning phase in the first 3–5 visit sessions) in Max. Gabor count, Supp. Index and logMAR acuity followed by a slow-learning phase that slowly reached asymptotic performance after sessions 3–5 (about 1300–2100 trials perceptual learning), which are consistent with previous reports regarding the pattern of perceptual learning with fast- and slow-learning phase in amblyopia^59–61^.

#### Relation between task-related selective visual attention and interocular suppression/visual acuity across perceptual learning sessions

**Figure 6:**
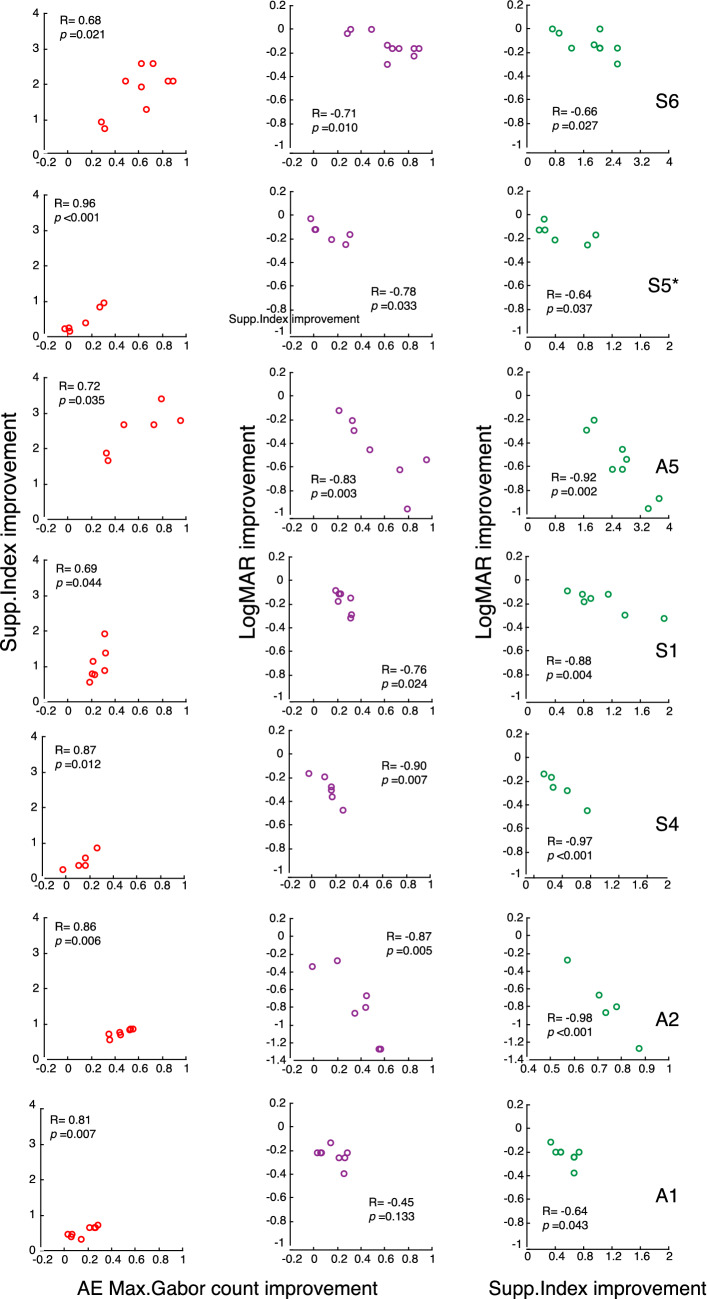
Relation of improvements in Max. Gabor count, Supp. Index and logMAR within-subjects across perceptual learning sessions. Correlation of the improvement in Max. Gabor count with the improvements in Supp. Index (**A**) and logMAR acuity (**B**) in the amblyopic eye of each individual participant across perceptual learning sessions re-plotted from the data in Fig. S2. (**C**) Correlation of Supp. Index with logMAR acuity re-plotted from the data in (**A**) and (**B**). The R values shown in each panel indicate Pearson’s coefficients. Correlation of the improvement in Max. Gabor count in the fellow eye with the improvements in Supp. Index and logMAR acuity across perceptual learning sessions is shown in Supplementary Information (Fig. S3).

We calculated the relative improvement in each parameter defined by (post-training value − pre-training value)/pre-training value, and conducted Pearson’s coefficient analysis. As seen in Fig. 6, the improvement in Max. Gabor count performance in the amblyopic eye was significantly correlated with the improvements in Supp. Index (left column) and logMAR acuity (middle column) in the amblyopic eye in all 7 individual participants, except for the correlation between logMAR and Max. Gabor count for Participant A1 (bottom row, middle column), across perceptual learning sessions, suggesting a tight relationship between task-related selective attention and interocular suppression/logMAR acuity. We also observed a significant correlation between interocular suppression and logMAR acuity in each participant (right column), which is consistent with previous reports^4,42,64^.

Note that the searching/counting performance in the fellow eye also progressively improved with perceptual learning. This is expected because we arranged 10% of the trials with attention tasks in the fellow eye. To demonstrate the correlates of task-related selective attention improvement in the fellow eye and Supp. Index/logMAR acuity, we plotted the improvements in Supp. Index and logMAR acuity against with the improvement in Max. Gabor count performance from the fellow eye in Fig. S3 (middle and right columns) with individual participants in Supplementary Information. We observed significant correlation between Max. Gabor count performance of the fellow eye with Supp. Index (middle column) and logMAR acuity (right column) only in 3 out of 7 participants (S6, A5 and S4), as compared to all 7 participants who showed significant correlations of Max. Gabor count performance of the amblyopic eye with Supp. Index and with logMAR acuity (Fig. 6).

#### The reduction of task-related selective attention bias towards the fellow eye is correlated with reduction of interocular suppression and improvement of visual acuity

**Figure 7:**
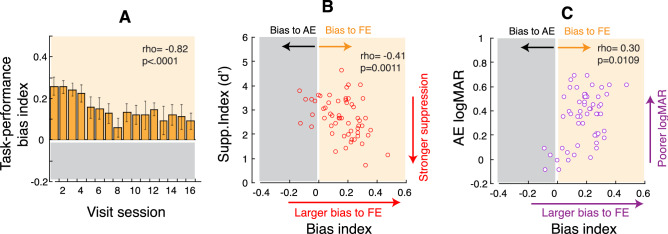
Correlation of task-performance bias index and Supp. Index and logMAR acuity. (**A**) Averaged task-performance bias index across 7 participants from the data in Supplementary Information (Fig. S4) correlated with perceptual learning sessions. Error bars denote SEM. Correlation of task-performance bias index with Supp. Index (**B**) and with logMAR acuity of the amblyopic eye (**C**) summarized from individual data in Fig. S4. The yellow area indicates attentional allocation bias towards the fellow eye; the gray area indicates attentional allocation bias towards the amblyopic eye. The correlations were tested by Spearman's coefficient because of non-parametric data with bias index.

With our experimental design, we expected to gain a greater attention deployment improvement in the amblyopic eye than in the fellow eye to reduce the habitual attention allocation bias to the fellow eye^42,49^. As task performance improved in both eyes (Figs. S2 and S3, left column), to take into account task-performance improvement in the amblyopic eye r*elative* to the fellow eye, we calculated “task-performance bias index”, defined as (FE performance − AE performance)/FE performance, across perceptual learning sessions, which are shown in Fig. 7. This bias index basically represents the difference in the performances between the eyes. Since the fellow eye always performs better than the amblyopic eye as reported in previous studies^56,58^, this index usually shows a positive number. We may also consider this bias index as an index of task-related selective attention bias between the eyes due to our high-attention demand tasks: if the index is positive, it suggests attentional allocation bias towards the fellow eye (Fig. 7A, data in yellow area); if the index is negative, it suggests attentional allocation bias towards the amblyopic eye (Fig. 7A, data in gray area). As seen in Fig. 7A, the performance bias index averaged across 7 participants progressively reduced with perceptual learning sessions. The individual participants’ data that contributed to Fig. 7A are shown in Supplementary Information (Fig. S4). As shown in Fig. S4, among 7 participants, 6 (with marginal significance in Participant S6) showed a significant negative correlation of bias index with perceptual learning sessions, indicating that the task-related selective attention bias towards the fellow eye progressively reduced with perceptual learning.

Furthermore, we observed a negative correlation between task-performance bias index and Supp. Index (Fig. 7B) and a positive correlation between bias index and logMAR acuity (Fig. 7C), suggesting the larger bias index (more towards to the fellow eye) the stronger interocular suppression and the poorer logMAR acuity. Figure 7B,C plot the correlation of task-performance bias index with Supp. Index (Fig. 7B) and with logMAR acuity (Fig. 7C) summarized from the individual data in Fig. S4. In the case of negative bias index (bias towards to the amblyopic eye), as seen in Participant A5, S1, and A2 in Fig. S4, these participants had the least suppression represented by the highest Supp. Index (*d*′) and the best visual acuity represented by the lowest logMAR. This relationship is also clearly evident in the summarized data in Fig. 7B,C. These correlations indicate that the larger attention allocation bias to the fellow eye, the greater interocular suppression and the worse visual acuity, suggesting a tight relationship between selective visual attention and amblyopic suppression.

Note that these participants had multiple measurements of Supp. Index and logMAR acuity that might have affected the improvement because of practice effects. To determine whether repeated measurements affected the training outcomes, we plotted the improvements in both suppression and logMAR acuity against the number of measurements. If there is an effect, there should be a trend for the improvement to increase with the number of measurements, or equivalently a greater improvement in the participants who had repeated measurements compared to those who were only measured pre- and post-training. Note that the training outcomes in participants who had repeated measurements could have been due to practicing both counting tasks and repeated measurements of Supp. Index and logMAR. However, we did not see such an effect, as shown in Fig. S5 in Supplementary Information. While some participants with repeated measures had the largest improvements, others had the least improvements, particularly for those with the largest number of repeated measurements. There was no correlation between repeated measures and improvements in both Supp. Index (left panel, R = 0.29, p > 0.05) and logMAR (right panel, R = 0.09, p > 0.05). Also, there was no significant difference between the groups of pre/post measure and repeated measure in both Supp. Index (p > 0.05) and logMAR (p > 0.05).

## Discussion

The findings from the present study are, to our knowledge, the first demonstration of the relationship between selective visual attention and interocular suppression in amblyopia and its role in the success of amblyopic training. We demonstrated that perceptual learning with dichoptic attention tasks improves attentional modulation of neural populations in V1 and IPS and reduces interocular suppression along with improvement of visual acuity in human adults with both anisometropic and strabismic amblyopia. And more importantly, we found that the reduction of interocular suppression significantly correlated with the improvement of selective visual attention in both training related and unrelated tasks in the amblyopic eye relative to the fellow eye.

### Evidence of relation between attention allocation bias between the eyes and interocular suppression in amblyopia

Our current findings provided further evidence suggesting a relationship between interocular suppression and selective visual attention in the amblyopic visual system. In contrast to previous work^42,56^, the novel finding in the present study is that the reduction of interocular suppression by training significantly correlated with the improvement of selective visual attention in both training *related* (Fig. 3B, left panel) and *unrelated* (Fig. 3B, right panel) tasks in the amblyopic eye *relative* to the fellow eye, suggesting a relation between *attention allocation bias between eyes* and interocular suppression. Strikingly, this relationship was further confirmed by the correlation analysis of task-related selective visual attention and interocular suppression across perceptual learning sessions within-subjects (Fig. 7 and Fig. S4). The significance of correlation analysis between the improvement of attentional modulation in V1, but not IPS, and Supp. Index is likely because V1 neurons receive monocular inputs, while the higher-level cortex (e.g., IPS neurons), receives binocular inputs that do not reflect eye-specific function. We arranged 90% of the trials with high-attention demand tasks in the amblyopic eye and only 10% of the trials with attention tasks in the fellow eye in perceptual learning. We expected to induce a greater improvement in selective attention to the amblyopic eye than to the fellow eye, and thus to reduce the habitual attention allocation bias to the fellow eye that is typically found in strabismic amblyopes^42,49^. Indeed, we observed a greater attention deployment improvement in the amblyopic eye than that in the fellow eye in both training-related (Fig. 2B,C) and unrelated (Fig. 2D) attention tasks. To take into account the effect of the improvements from both eyes in selective attention, we calculated the relative improvement in attention between the eyes and found a consistent correlation between the reduction of interocular suppression and the improvement of selective attention in both training related and unrelated tasks. Our results demonstrated the more selective attention improvement to the amblyopic eye, the greater the reduction of interocular suppression (Fig. 3B, the left and right panels). The correlation analysis across perceptual learning sessions within-subjects further confirmed the relationship between interocular suppression and attention allocation bias between eyes, which is summarized in Fig. 7: the larger attention allocation bias to the fellow eye, the greater interocular suppression (Fig. 7B) and the worse logMAR acuity (Fig. 7C).

### Comparison with previous amblyopic training studies in alleviating amblyopic suppression

In contrast to the majority of behavioral training studies in amblyopia that have focused on whether training improves visual acuity and/or stereoacuity, as seen in recent review articles^16,65^, the present study focused on exploring the relationship between selective visual attention and amblyopic suppression and its role underlying the neural basis of successful anti-suppression training in amblyopia. We designed our dichoptic perceptual learning stimuli, which avoided typical contrast sensitivity-based tasks and required significant attentional efforts from the amblyopic eye. Our training tasks were based on the attention framework^40,45,66,67^, in which we arranged “targets” in the amblyopic eye and “distractors” in the fellow eye in 90% of the trials. This arrangement had reversed the habitual (natural) viewing condition in amblyopia, since the fellow eye commonly leads selective visual attention to search for targets. With the arrangement of “targets” and “distractors” in the different eyes, we hoped to assess their particular effects^40,67^, as demonstrated in a recent fMRI study, in which the “distractors” induced suppression while the “targets” induced facilitation in visual processing of the cortex^66^. Regarding the arrangement of “targets” and “distractors” in the different eyes, the dichoptic training or videogame play in previous studies^22,26,32,62^ assembled a similar arrangement to ours: for example, falling objects (targets) in the amblyopic eye and ground plane objects (distractors) in the fellow eye were used in the dichoptic Tetris videogame play^22,26^. With this arrangement, previous studies demonstrated the same or larger magnitude of visual acuity improvement after just 5 h^26^ or 10 h^22^ of videogame play, compared to 40 h of monocular videogame play^27^. Our results are comparable and consistent with the Li et al.^22^ study in adult amblyopes, in which most participants showed visual acuity improvement and reduction of interocular suppression in the fast-learning phase around 3–5 visit sessions (6–10 h training), followed by continuing improvement in the later slow-learning phase, as shown in Fig. S2 for some participants. It appears that dichoptic training, particularly with the arrangement of “targets” in the amblyopic eye and “distractors” in the fellow eye, was more effective than monocular training. Besides, our study demonstrated the same training outcomes in two groups with balanced and equal contrasts in the eyes (Fig. 5), indicating the effectiveness of perceptual learning using top-down attention tasks (equal contrast strategy). The fact that dichoptic attention task itself without balancing the contrasts in the eyes resulted in the same amount of reduction in interocular suppression as with balanced contrast strategy shown in the current study strongly suggests a role of selective visual attention in amblyopic suppression.

On the other hand, strengthening bottom-up excitatory connections by increasing contrast to the amblyopic eye presumably benefits the recovery of latent binocular capacity and the reduction of suppression, as found in previous studies^22,26,32,62^. Thus, we expected to see the additive effect in balanced contrast strategy (e.g., a greater improvement in balanced contrast strategy than in equal contrast strategy). However, such additive effect was not observed in various training outcomes that were compared in Fig. 5. This might be due to: (1) small sample size in the present study to reveal the differences, (2) unequal number of participants in the Aniso and Strab groups or, (3) the high-demand attention tasks that were included in both equal contrast and balanced contrast strategies. It is possible that the training outcomes driven by the additional bottom-up stimulus salience factor (increasing contrast to the amblyopic eye) were overridden by the high-demand attention tasks, because the dichoptic attention task itself without balancing the contrasts in the eyes resulted in similar training outcomes to those with balancing the contrasts, as demonstrated with the individual data from the participants in Fig. 5. However, whether there is an additive effect in the balanced-contrast strategy with dichoptic high-demand attention tasks in training needs to be confirmed with a greater number of participants in further studies.

Our study found a significant correlation between the reduction of interocular suppression and the improvement of visual acuity across subjects (Fig. 4B) and within-subjects (Fig. 7C), which is consistent with our previous study^42^ that used the same method to quantify interocular suppression in adults with amblyopia. Our finding is also consistent with the study^64^ in the correlation of visual acuity and interocular suppression with a large data sample (n = 106) that combined multiple studies and used contrast balance point with a dichoptic motion coherence task^68^ to quantify interocular suppression. However, interocular suppression was not always found to correlate with visual acuity^5,62^. The discrepancies might be due to different methods that were used to quantify interocular suppression, since there is no gold standard for suppression measurement in both clinic and research. Nevertheless, our study and previous dichoptic training studies with contrast balance strategy^22,32,62^ resulted in reduction of interocular suppression in adults with amblyopia.

### Implications and future study

The significant correlation of attention allocation bias between eyes and interocular suppression found in the present study has important implications. Under binocular viewing, normal vision has no attention allocation bias between eyes; however, attention is allocated preferentially toward the non-amblyopic fellow eye in strabismic amblyopes^42,49^. As demonstrated in the present study, training with dichoptic attention tasks reduced such attention allocation bias between the eyes (Fig. 7A), which correlated with the reduction of interocular suppression (Fig. 7B). Based on our findings, we propose that attention allocation bias between eyes might be related to visual suppression of input to the amblyopic eye. It is plausible that amblyopia (decreased visual acuity in the non-fixating eye) might be a consequence of long-term attention allocation bias to the fixating eye in strabismus. Reducing visual acuity in the non-fixating eye (to be amblyopic) may relieve attentional effort from the fixating eye to overcome diplopia caused by strabismus. More importantly, this proposal may lead to a broader implication in which selective visual attention may modulate interocular suppression through top-down attentional processes in normal and amblyopic vision, which potentially provides a broader context than the traditional concepts regarding amblyopia and interocular suppression in general. Our findings from the present study may lead to a subsequent confirmatory research on identifying the neural correlates of how top-down processes modulate interocular suppression in visual cortical areas, including V1 and extra-striate cortex, which remain poorly understood. We want to point it out that some of the participants had multiple measures of Supp. Index and logMAR, which might have an effect on the improvements that cannot be distinguished between the effects of the attentional task and repeated measures. In addition, it is possible that the attention improvements due to training with our stimuli might have occurred without the effect of attentional cue. We cannot provide a definitive answer because we did not test cue validity in the current study. As we have mentioned in “Introduction”, it is not clear whether and how much the cue contributed to attentional selection (the trained eye) without using both valid and invalid cues. The role of the cue per se remains to be shown in future studies in experiments that manipulate the validity of the cue.

## Conclusions

The present study explored the relationship between selective visual attention and amblyopic suppression and its role in the success of amblyopic training. The study demonstrated a significant correlation between the reduction of interocular suppression and the improvement of selective visual attention in both training related and unrelated tasks in the amblyopic eye relative to the fellow eye in perceptual learning, suggesting a relation between interocular suppression and selective visual attention. Furthermore, this study also demonstrated the translational benefit from the new insights of basic science to clinical therapies in amblyopic treatment. Training with a dichoptic approach that incorporates attention demand tasks in the amblyopic eye is an effective way of treating amblyopia.

## Methods

### Participants

Seventeen adults (8 males) with amblyopia (6 anisometropic and 7 strabismic or mixed anisometropic/ strabismic) and with normal-vision controls (n = 4) participated in the study. The age range was between 22 and 66 years (mean ± SD, 45.3 ± 14.35). Amblyopia was defined as anisometropic amblyopia (≥ 1 D of refractive error interocular difference; referred to as “Aniso”), strabismic amblyopia, or a mix of both. We refer to strabismic or mixed amblyopia as “Strab” in this study. Inclusion and exclusion criteria for amblyopia can be found in Supplementary Information (Methods). Clinical information is provided in Table 1. The study protocol was approved by The Smith-Kettlewell Institutional Review Board and conformed to the tenets of the Declaration of Helsinki. Informed consent form was obtained from all the participants after the experimental procedures were explained. The authors were attesting that the participants were aware of the study purpose, risks and benefits.

**Table 1:** Clinical details of the participants with amblyopia.

Participant ID	Diagnosis	Age	Gender	Visual acuity^‡^ (logMAR)		Stereoacuity^‡^	Refractive errors		Deviation	History
				Fellow eye	Amblyopic eye		Fellow eye	Amblyopic eye		
A1	A	27	f	0.04	0.46	200″	− 7.00 + 0.50 × 90	− 8.00 + 1.25 × 40	Ortho	No patching
A2	A	22	m	− 0.09	0.30	200″	+ 1.25 + 0.75 × 70	+ 3.25 + 0.50 × 110	Ortho	Patching done
A3*	A	52	f	0.04	0.50	100″	− 1.75 + 1.00 × 10	− 5.00 + 0.75 × 160	Ortho	Patching done
A4	A	51	f	0	0.50	200″	− 0.25	+ 3.00 + 0.50 × 90	Ortho	Patching done
A5	A	49	f	0	0.24	70″	0.50 + 0.50 × 90	− 1.50 + 2.25 × 130	Ortho	Patching done
A6	A	50	f	− 0.2	0.38	200″	− 1.00 + 0.50 × 30	+ 4.5 + 0.50 × 150	Ortho	Patching done
S1	S&A	59	m	− 0.04	0.70	n/a	+ 0.75	− 1.00 + 0.75 × 25	XT 14, L/R 4	Surgery and patching
S2*	S	38	m	− 0.09	0.30	n/a	− 1.50	− 1.50	XT 12, R/L 4	Patching done
S3	S&A	40	f	0	0.518	n/a	+ 3.25 + 2.00 × 170	Plano	XT 8	Surgery and patching
S4	S&A	62	m	0	0.70	n/a	+ 1.50 + 0.50 × 40	+ 6.25 + 1.25 × 40	XT 4, R/L 2	Patching done
S5*	S&A	66	f	− 0.02	0.46	n/a	+ 1.25 + 1.00 × 105	+ 3.50 + 2.25 × 85	XT 8	Surgery and patching
S6	S&A	28	m	− 0.09	0.62	400″	Plano	+ 1.00 + 0.50 × 90	ET 6	No patching
S7*	S&A	55	F	0	0.70	n/a	− 2.25 + 1.25 × 90	− 4.75 + 1.00 × 110	XT 20	No patching

*A* anisometropic amblyopia, *S* strabismic amblyopia, *S&A* mixed strabismus and anisometropia, *m* male, *f* female. Deviation at near (33 cm) with best optical correction is shown in prism diopters. *XT* exotropia, *ET* esotropia, *L/R* left-eye hypertropia, *R/L* right-eye hypertropia. *Indicates that the participant did not complete 16 visits. ^‡^ Indicates an entrance level of logMAR acuity and stereoacuity. *n/a* indicates the participants who had non-measurable stereoacuity.

### Perceptual learning

The details of stimulus display and design can be found in Supplementary Information.

#### Perceptual learning strategy and procedure

Using balanced contrasts to train amblyopia results in reduction of interocular suppression and improvement of visual acuity in adults with amblyopia^22,23,31,32^. To be comparable with previous studies, the present study also used balanced contrast strategy in one group of participants, and expected to compare the training outcomes with those from another group of participants who underwent dichoptic training with equal contrasts in the two eyes. Therefore, our participants were assigned into two groups with different strategies and procedures in perceptual learning across sessions while performing the same tasks (searching/counting): (1) group with equal contrasts in the two eyes (35% contrast in each eye) across perceptual learning sessions, in which the level of attention required was incremented by progressively decreasing stimulus presentation duration from the initial session at 1000 ms to the end session at 200 ms; (2) group with balanced contrasts in the eyes, in which the contrast of the amblyopic was increased for equal perceptual visibilities in the two eyes across perceptual learning sessions, while the stimulus duration remained the same, either 200 ms across all visit sessions or 350 ms across all visit sessions, according to the initial stimulus duration used for the participant. In this group, the level of attention required was incremented by decreasing contrast of the stimuli in the amblyopic eye at the “contrast balanced point”, as previous studies have reported that the contrast balanced point is typically reduced after the training by using balanced contrast strategy^22,23^. The details of how to balance the contrasts between the eyes are described in Supplementary Information under the subtitle of “Contrast matching between the two eyes for equal perceptual visibilities in balanced contrast group”.

All participants from the two training groups, equal-contrast and balanced-contrast, performed the tasks under the best optical correction at the distance of 85 cm and came in for about 2 visits (sessions) per week, 2 h per visit, for 2 months (in total of 16 visits, ~ 7000 trials of repetitive practice). This amount of training was chosen because after 7000 trials, observers have shown no further improvement^61^. Among 13 participants, 4 participants did not complete 16 sessions (~ 7000 trials), which were marked in Table 1. Among these participants, S2 completed 9 sessions (~ 3900 trials); S5 completed 6 sessions (~ 2600 trials); S7 completed 8 sessions (~ 3500 trials); A3 completed 12 sessions (~ 5250) trials.

### Attention deployment evaluation before and after perceptual learning

#### Attention deployment in trained tasks

As our perceptual learning stimuli included high attention-demand tasks, they were expected to induce attention deployment improvement. Thus, the task-performance itself was also considered to represent attention deployment. To be consistent and comparable between participants for the training, we used the same stimulus conditions (35% contrast in each eye and 200 ms stimulus duration) for all participants to evaluate attention deployment changes with trained tasks before and after perceptual learning. These evaluations are plotted in Fig. 2.

We had two ways to quantify attention deployment in trained tasks in each eye. Firstly, a Weibull cumulative distribution function with additional scaling coefficient and constant offset (Eq. 1) was used to fit the data (Fig. 2A,B). Fitting these functions was done in KaleidaGraph to extract the *η* and *β* parameters.

1 $${\mathbf{y}} = \left\{ {1 - \exp \left[ {\left( {\frac{ - {\mathbf{x}}}{\eta }} \right)^{\beta } } \right]} \right\}\Delta_{N} + N_{0}$$

where *η* is a semi-saturation constant, *β* measures how fast the function rises, Δ_*N*_ is the amplitude scaling, and *N*_*0*_ is the constant offset. The coefficient of determination (R^2^) was used to assess goodness of the fit. The parameters *η* and *β* were compared pre- and post-learning.

Secondly, we used the searching/counting performance at a given maximum number of Gabor-set (Max. Gabor count) to evaluate attention deployment changes with trained tasks (Figs. 2C, 6, 7; Figs. S2, S3 and S4), because a previous study has shown that feature counting, particularly when the number of features is beyond 5, represents fundamental components of attention^46^.

#### Attention deployment in untrained tasks

##### Stimuli and display

We used a different attention task, which was unrelated to the training task, to evaluate attention deployment changes because of training with our dichoptic attention tasks. Each amblyopic participant had structural and functional MRI scans conducted on a 3 T Tim Trio scanner (Siemens, Munich, Germany) before perceptual learning, which allowed us to be able to use EEG sourced imaging to evaluate attentional modulation of population neurons in specific cortical areas. We measured and compared attentional modulation in IPS and V1 using source-imaged Steady-State Visual Evoked Potentials (SSVEP) before and after the training for 10 out of 13 participants (Participants A2, S6 and S7* did not complete this measurement after the training). The measurement and selective attention tasks along with the stimulus display, EEG data acquisition and source localization in V1 used in the present study were identical to those used in our previous study^42^. The functional areas IPS were defined using multi-subject probabilistic atlases created by aligning individual subject data to a standard space^69^. SSVEP was measured in response to two sinusoidal gratings flickering on and off at different temporal frequencies (Left eye: F1 = 16.67 Hz; right eye: F2 = 12.5 Hz), which allowed for frequency tagging of EEG signals. A cue indicated the grating to attend (left or right), and the participant was instructed to attend to the grating on the cued side while maintaining fixation in the center, and to report the presence or absence of a contrast increment on the cued grating during EEG recordings. The contrast increment was adjusted to be at the threshold level for each eye of the participants. Thus, the attended and ignored responses were compared on identical stimuli.

##### ROI-based analysis and attention index

A discrete Fourier transform was used to estimate the response amplitude at the stimulation frequencies for each participant. The first harmonic component of the stimulus-driven frequencies (16.67 and 12.5 Hz) was analyzed. To take into account the different noise levels for each participant^70^, we computed the signal-to-noise ratio (SNR) by dividing peak amplitudes by the associated noise, which was defined for a given frequency by the average amplitude of the two neighbor frequencies (stimulus frequencies ± 0.5 Hz). The details of ROI-based analysis were reported in our previous study^42^. We calculated “attentional modulation” of population neurons in areas V1 and IPS from visual input of each eye, which was defined as the *difference between the attended and ignored SNRs*, as shown in Figs. 2D and 3B.

### Interocular suppression measurement before and after perceptual learning

We measured interocular suppression for all 13 participants pre- and post-training. As the training spanned a period of time, we had multiple interocular suppression measurements across perceptual learning sessions for only for 7 out of 13 participants, which allowed us to track the reduction of interocular suppression across the training sessions. The stimuli, display and procedures to measure interocular suppression were identical to those used in our previous study^42^. In brief, a horizontal and/or a vertical rectangle (1 cpd in spatial frequency, 35% contrast) ranging in size from 0.23° × 0.94° to 1.64° × 5.16° was presented to one eye (monocular condition, 50% of the trails) or each eye (binocular condition, 50% of the trails) within a block. Rectangle size was adjusted so that its orientation was identified correctly on 75% of the trials when presented monocularly to the amblyopic eye. The tasks for the participants were to report how many rectangles they saw (one or two) and, if they saw only one, to report its orientation. If the participant reported both targets in the binocular condition, the response was counted as a hit; if the participant reported two rectangles when only one rectangle was presented (monocular condition), the response was counted as a false alarm. Suppression index (referred to as “Supp. Index”) was defined as *d*′ = z (hits) − z (false alarms). As the binocular condition trials measure suppression by determining whether the rectangle presented to the amblyopic eye is suppressed when a rectangle is presented to the fellow eye, therefore the Supp. Index (*d*′) represents the degree of interocular suppression. A smaller *d*′ represents less binocularity and indicates stronger interocular suppression.

### Visual acuity and stereoacuity evaluation before and after perceptual learning

Visual acuity was measured at 6 m with a logMAR chart (Bailey–Lovie) at the best optical correction. Stereoacuity was evaluated with the Random-Dot Stereo Butterfly card (Stereo Optical, Chicago, IL, USA) at 40 cm with the best optical correction.

### Statistical analysis

Significant differences in logMAR acuity before the training between amblyopic subgroups and between training groups were identified by a Mann–Whitney Test for two independent samples. Significant differences in fit parameters (Fig. 2B), Max. Gabor count performance (Fig. 2C), count error (Supplementary Information, Fig. S2), Supp. Index (Fig. 3) and logMAR acuity (Fig. 4) between pre- and post-perceptual learning were identified by a Wilcoxon Signed-Rank Test for paired samples. Significant differences between perceptual learning improvement and pre- training baseline (Figs. 2, 3 and 4) were identified by a Wilcoxon Signed-Rank Test for a single sample. Significant differences between groups and subgroups (Figs. 2, 3 , 4 and S5) and between training groups (Fig. 5) were identified by a Mann–Whitney test for two independent samples. The Bonferroni correction was used to control the familywise error rate for multiple comparison, with a significance level set to 0.05/3 = 0.0166. The normality of data was tested by Shapiro–Wilk Test for the data in Fig. 6 and Figs. S2 and S3 (*p* > 0.05). Correlation coefficients and significances were calculated using one-tailed Pearson's R in Fig. 6, Figs. S2 , S3 and S5, and with Spearman’s *rho* in Figs. 3B, 4B, 7 and Fig. S4. These tests were conducted using the Real Statistics Resource Pack software in Excel (Copyright: 2013–2020, Charles Zaiontz. http://www.real-statistics.com).

## Supplementary Information

Supplementary Information.

## Data Availability

All data generated or analyzed in this study are included in this published article and its Supplementary Information file.

## Abbreviations

PL: Perceptual learning
Strab: Strabismic amblyopia
Aniso: Anisometropic amblyopia
AE: Amblyopic eye
FE: Fellow eye
IPS: Intraparietal sulcus
V1: Primary visual cortex
