# Author Correction: Perceptual learning with dichoptic attention tasks improves attentional modulation in V1 and IPS and reduces interocular suppression in human amblyopia

**DOI:** 10.1038/s41598-022-15265-9

**Published:** 2022-06-27

**Authors:** Chuan Hou, Spero C. Nicholas

**Affiliations:** The Smith-Kettlewell Eye Research Institute, San Francisco, CA 94115 USA

Correction to: *Scientific Reports* 10.1038/s41598-022-13747-4, published online 11 June 2022

The original version of this Article contained an error in the y-axis label of Figure 4(C), where “stereoacuity improvement (%)” should read “stereoacuity improvement”.

The original Figure 4 and accompanying legend appear below.

**Figure 4:**
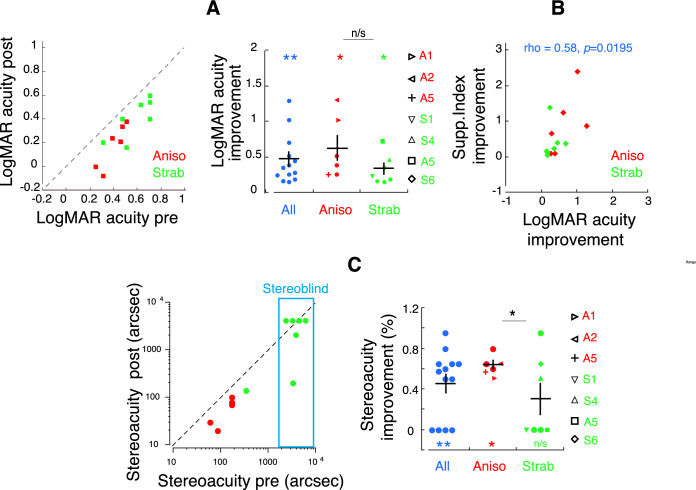
Visual function improvement after perceptual learning (PL). (**A**) LogMAR acuity pre- and post-PL. Data below the dashed line (1:1 ratio of logMAR acuity pre- and post-PL) indicate logMAR improvement. (**B**) Correlation between improvements of logMAR and Supp. Index (*d*′). (**C**) Stereoacuity pre- and post-PL. Data below the dashed line (1:1 ratio of stereoacuity pre- and post-PL) indicate stereoacuity improvement. Colors denote the group. Error bars denote SEM. Note that in (**C**), data with non-measurable stereoacuity were plotted as 4000 arcsec, shown in blue box. *, and ** denote p < 0.05 and p < 0.01, respectively. Black asterisks indicate paired tests and colored asterisks indicate one- sample tests. Participants who were tested repeatedly across PL sessions in “Results” section “Correlation of task-related selective attention and interocular suppression/visual acuity across perceptual learning sessions” were identified by different symbols in the right panels of (**A**) and (**C**).

The original Article has been corrected.

