# Supplementary material for: Perceptual learning with dichoptic attention tasks improves attentional modulation in V1 and IPS and reduces interocular suppression in human amblyopia
Source: Sci Rep. 2022 Jun 11;12:9660. doi: 10.1038/s41598-022-13747-4 (PMC9188564; doi:10.1038/s41598-022-13747-4)
Supplement: Supplementary file 1 — Supplementary Information. [file 41598_2022_13747_MOESM1_ESM.docx]

**sSupplementary Information:**

**Perceptual learning with dichoptic attention tasks improves attentional modulation in V1 and IPS and reduces interocular suppression in human amblyopia**

Chuan Hou* and Spero C. Nicholas

**I: Extended data for Fig. 2 in manuscript: Searching and counting errors pre- and post-perceptual learning**

As shown in Fig S1, searching and counting errors (defined as the difference between displayed and reported number of Gabors) decreased after perceptual learning (PL).

**Insert Fig. S1 about here**

**
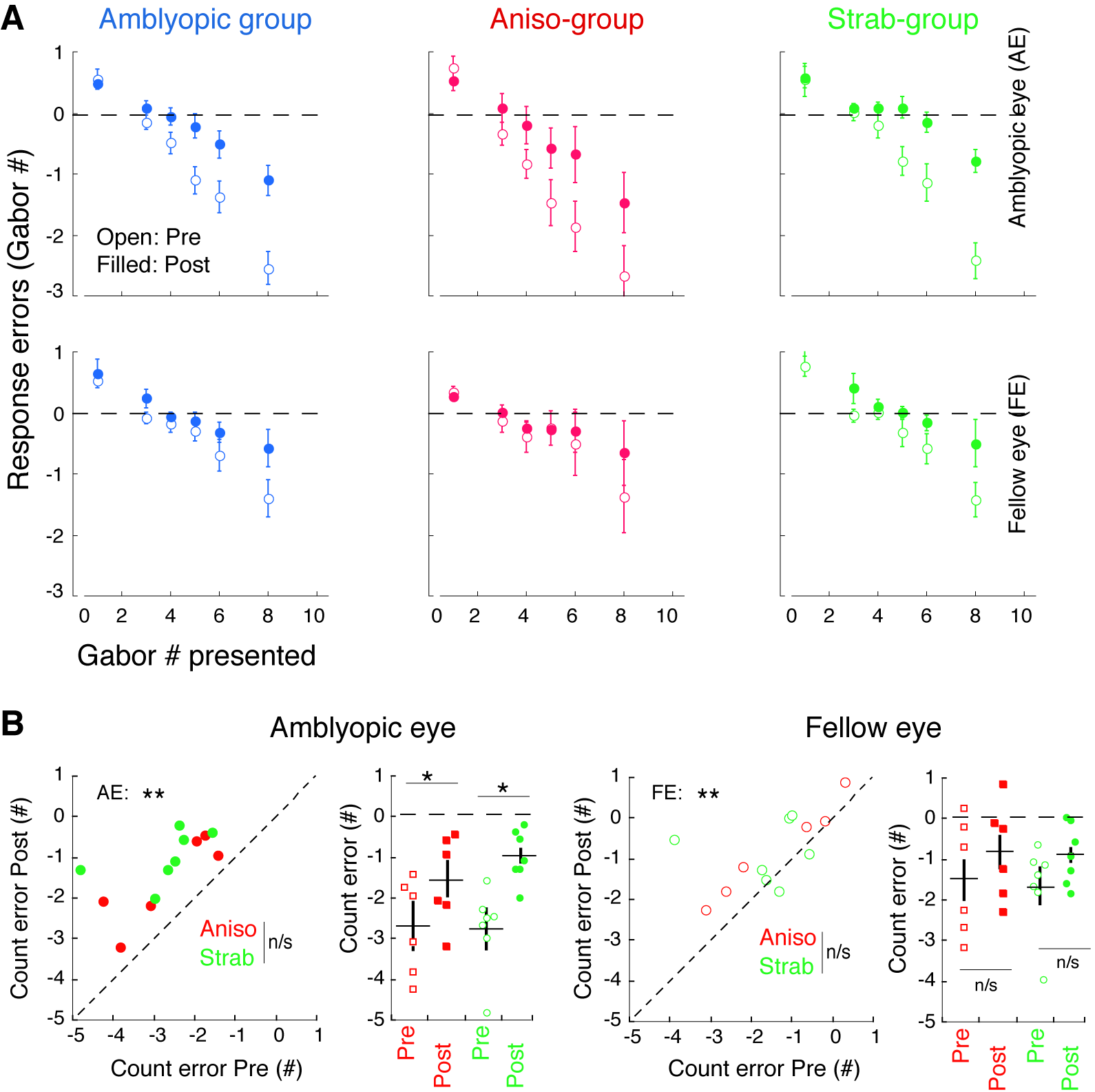
**

**Figure S1.** Extended data for Fig. 2. Searching and counting errors pre- and post-perceptual learning (PL). (A) Group mean of searching and counting errors. Colors denote the group. Error bars denote SEM. The horizontal dashed lines indicate 0 error where the responded Gabor # equals the presented Gabor #. (B) Comparison of errors at the presented Max. Gabors pre- and post-PL. *, and ** denote p<0.05 and p<0.01.

**II:** **Visual acuity and stereoacuity improved after perceptual learning**

Visual acuity (logMAR) improved with perceptual learning. It is worth noting that the age (mean ± SD, 45.3 ± 14.35) of the participants in the current study was in general older than previous perceptual learning studies in adult amblyopia; most of our participants (8 out of 12) were between 49 and 66 years old. For example, the three participants in the Ooi et al. (2013) study ^1^ were 24, 26, and 38 years old, and the 18 participants in the Li et al. (2013) study ^2^ had a mean age 22.3 with range of 21-31 years. In general, younger individuals have more neural plasticity for recovering visual functions ^3,4^. It is encouraging for clinical application that visual acuity improvement was seen even in the participants around 60 years of age (S1=59, S4=62 and S5*=66) in our study. Note that two anisometropic amblyopes, Participant A2 (22 years old) and A5 (49 years old) reached normal visual acuity at the end of training at -0.08 logMAR (20/16) and 0 logMAR (20/20), respectively.

Stereoacuity improved in 9 out 13 participants with perceptual learning. Participants A3 and A5, who were anisometropic amblyopes, reached 20 and 30 arcsec at the end of training, respectively. This finding is consistent with a previous study ^5^, in which 3 out of 6 Anisos had fully stereoacuity recovery after monocular video-game play training. Notably, among 6 strabismic amblyopes who had non-measurable stereoacuity before the training, two strabismic participants (S3 and S4) had measurable stereoacuity of 200 and 2000 arcsec, respectively, at the end of training. Participant S7 gained fusion function in the third session of training, although she still had non-measurable stereoacuity. Two strabismic participants (S4 and S5*) reported short periods of seeing double during the first month of training, but then double vision disappeared after a month of training. Participant S1 reported sensing the depth of trees when walking in the park for the first time in his life. However, he still had non-measurable stereoacuity at the end of training. We think that some of these strabismic participants might have gained coarse stereoacuity that was beyond the range of current available stereoacuity measurements, e.g., Random-Dot Stereo Butterfly card that we used. Participant S3, who reached 200 arcsec at the end of training, watched a 3D movie and enjoyed depth perception for the first time in her life on the second month of training.

**III: Individual data in correlation of task-related selective attention and interocular suppression/visual acuity in *the amblyopic eye* across perceptual learning sessions**

As seen in Fig. S2 (left column), the searching/counting performance from the amblyopic eye, in general, progressively improved with PL sessions (i.e., reported number of Gabors was closer to displayed number of Gabors). For simplicity, we plotted and compared the task-performance of the amblyopic eye at Max. Gabors from the left column, highlighted in gray at 8 displayed Gabors, with Supp. Index (second column) and with logMAR acuity in the amblyopic eye (third column) as a function of PL sessions. We observed a positive relation of Max. Gabor count / Supp. Index and a negative relation of logMAR acuity along with PL sessions, suggesting that task-related selective attention and logMAR acuity progressively improved with PL sessions while interocular suppression decreased.

Furthermore, we also observed a pattern of initial rapid improvement (fast-learning phase in the first 3-5 visit sessions) in Max. Gabor count, Supp. Index and logMAR acuity followed by a slow-learning phase that slowly reached asymptotic performance after sessions 3-5 (about 1300-2100 trials perceptual learning). Participant S5*, who only completed 9 visit sessions, showed a clear fast-learning phase in Sessions 2-6. The profile of perceptual learning in our study is in agreement with the majority of previous perceptual learning studies, where perceptual learning generally follows fast- and slow-learning phases. Fast-learning usually happens within the first 2-4 sessions (roughly 1000-2000 trials) ^6-8^, quickly followed by a slow-learning phase that slowly reaches asymptote. After 7000 trials, naïve normal observers show no further improvement ^6^. The right column in Figure 7 is the re-plot from the second and the third columns, which demonstrates that visual acuity improves while interocular suppression reduces across training sessions.

**Insert Fig. S2 about here**


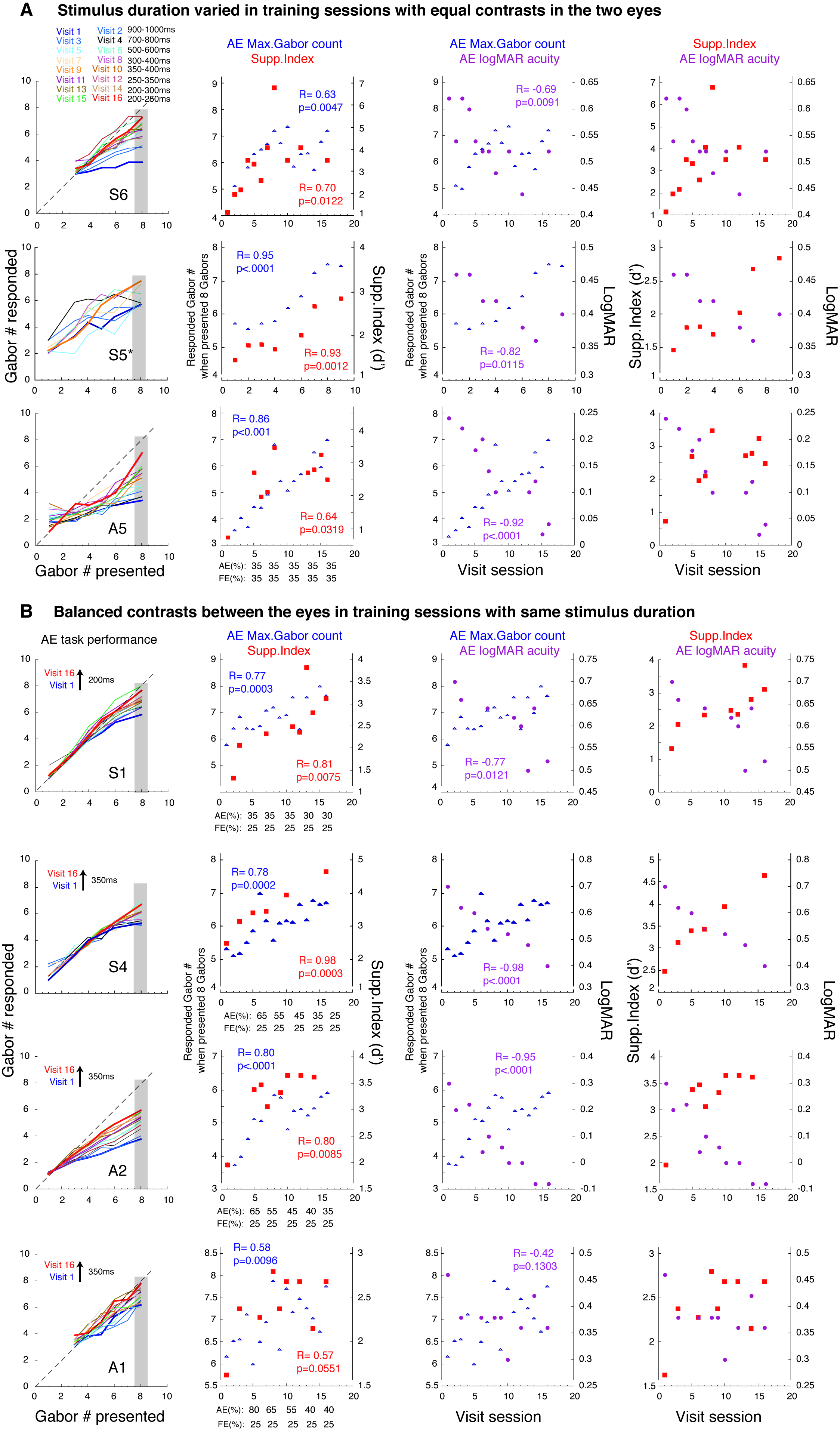


**Figure S2.** Task performance and correlates of Max. Gabor count, Supp. Index and logMAR acuity in the amblyopic eye (AE) with perceptual learning (PL) sessions. Participant ID is shown in the left column. (A) Equal contrasts between the eyes in training (35% contrast in each eye). The stimulus duration varied across PL sessions as shown in the top panel of the left column. (B) Balanced contrasts between the eyes in training. The contrast in each eye for each participant used in PL session was shown underneath the panel of the second column. Left column: AE task performance across PL sessions. Colors denote the averaged performances from each visit session, with the bold lines in blue as Visit 1 and in red as Visit 16. Note that Participant S4* only completed 9 sessions. Dashed lines indicate 1:1 ratio between reported and displayed number of Gabors. The gray rectangle highlights the Max. Gabor count when 8 Gabors were displayed. The Max. Gabor count was compared to Supp. Index (second column) and logMAR acuity (third column) across visit sessions. The right column re-plots Supp. Index and logMAR acuity from the second and third columns across PL sessions for a comparison. The R values shown in each panel of the second and the third columns indicate Pearson’s coefficients of Max. Gabor count in the AE (blue), Supp. Index (red) and logMAR acuity (purple) in the AE with PL sessions. Extended Data for Fig. S2 with the performances in the fellow eye are shown in Fig. S3, as seen below.

**IV: Individual data for correlation of task-related selective attention and interocular suppression/visual acuity in *the fellow eye* across perceptual learning sessions**

As shown in Fig. S3 (left column), the searching/counting performance from the fellow eye also improved with PL sessions in some participants (i.e., reported number of Gabors was closer to displayed number of Gabors). We observed significant correlation between Max. Gabor count performance of the fellow eye with Supp. Index (middle column) and logMAR acuity (right column) only in 3 out of 7 participants (S6, A5 and S4), as compared to all 7 participants who showed significant correlations of Max. Gabor count performance of the amblyopic eye with Supp. Index and with logMAR acuity (Fig. 6 in manuscript).

**Insert Fig. S3 about here**

**
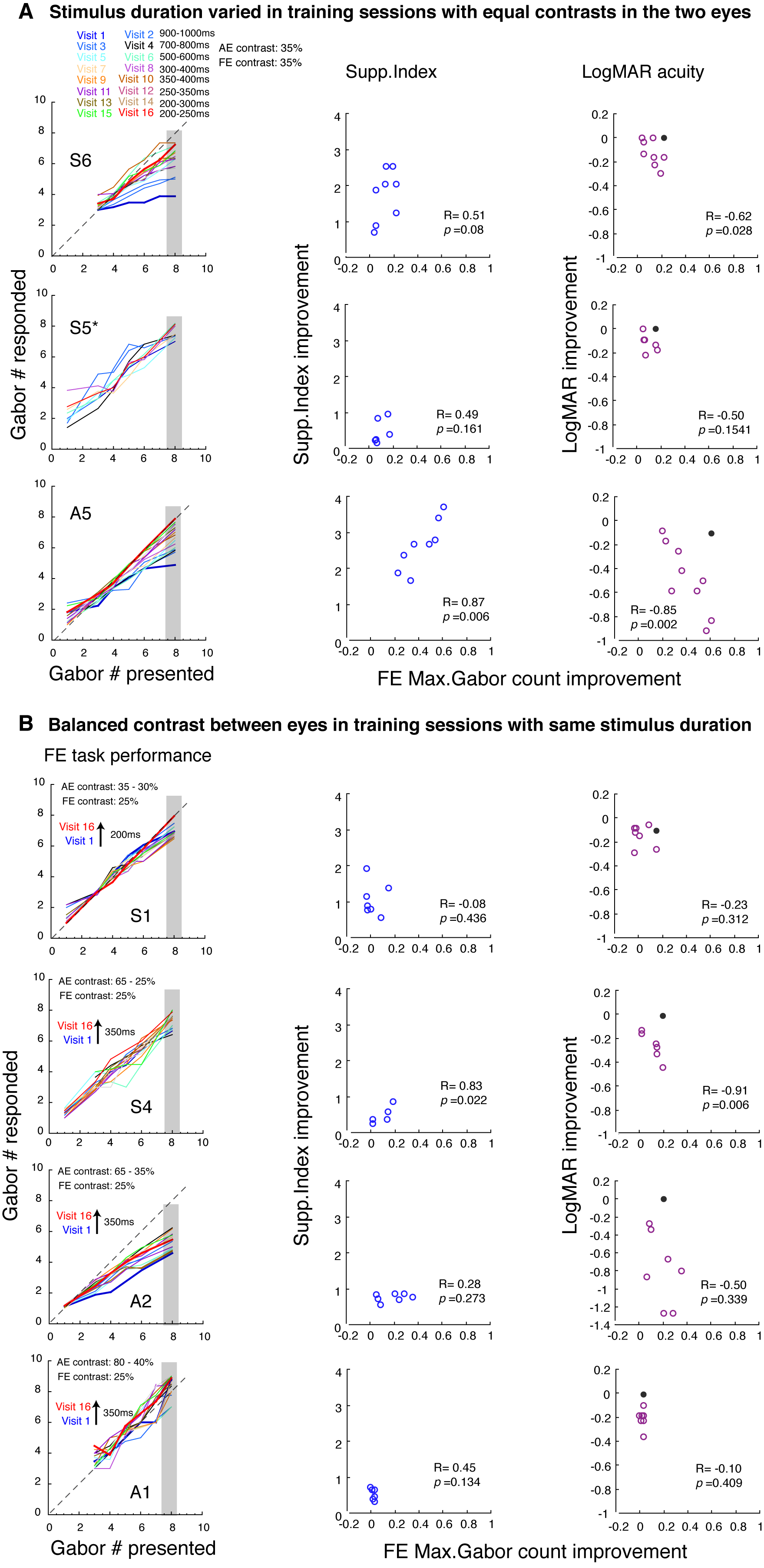
**

**Figure S3.** Task performance of the fellow eye (left column) and correlation of the improvement in Max. Gabor count in the fellow eye with the improvement in Supp. Index (middle column) and logMAR acuity (right column; open symbols: AE logMAR; filled symbols: FE logMAR) across perceptual learning (PL) sessions. (A) Equal contrasts between the eyes in training. (B) Balanced contrasts between the eyes in training. In the left column, colors denote the averaged performances from each PL session, with the bold lines in blue as Visit 1 and in red as Visit 16. Note that Participant S4* only completed 9 sessions. Dashed lines indicate 1:1 ratio between reported and displayed number of Gabors. The gray rectangle highlights the Max. Gabor count reported when 8 Gabors were displayed. Participant ID shown in each panel was matched with participant ID in Fig. 6 in the manuscript and Fig. S2 for the performance from the amblyopic eye. The R values shown in each panel of the middle and right columns indicate Pearson’s coefficients.

**V: Individual data in task-related selective attention bias towards the fellow eye reduced by perceptual learning**

As shown in Fig. S4A, task-related selective attention bias towards the fellow eye reduced with perceptual learning in 6 out of 7 participants (with marginal significance in Participant S6). Correlations of attention bias towards the fellow eye and Supp. Index / logMAR acuity within-subjects across perceptual learning are shown in Fig. S4B and C.

**Insert Fig. S4 about here**


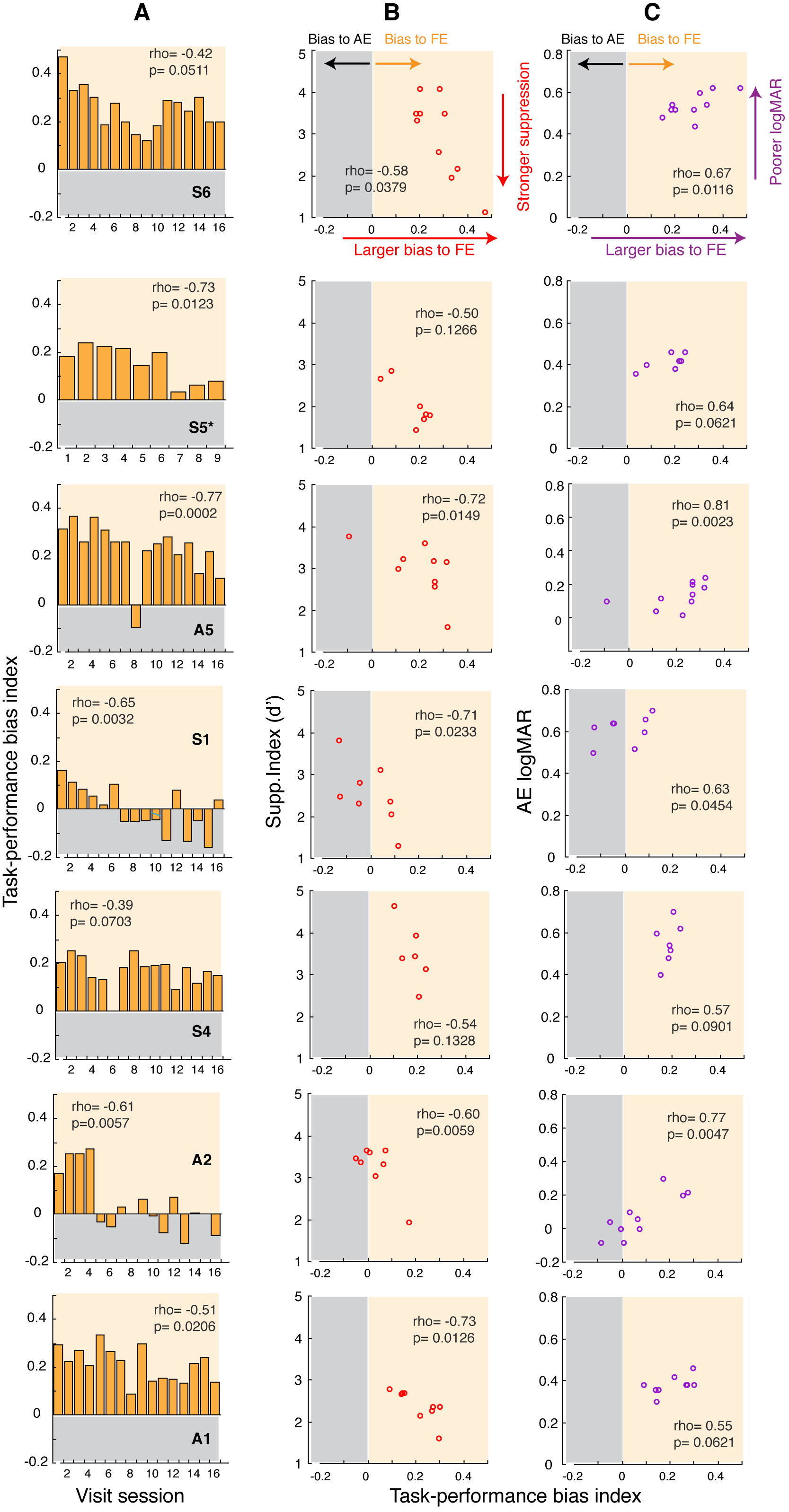


**Fig. S4.** Task-related selective attention bias towards the fellow eye reduced by perceptual learning. Individual data of task-performance bias index correlated with perceptual learning sessions (A), with Supp. Index (B) and with logMAR acuity (C). The yellow areas indicate attentional allocation bias towards the fellow eye; the gray areas indicate attentional allocation bias towards the amblyopic eye. Note that in the case of negative bias index (gray areas; bias towards to the amblyopic eye), these participants (Participant A5, S1 and A2) had the least suppression represented by the highest Supp. Index (*d’*) and the best visual acuity represented by the lowest logMAR. The correlations were tested by Spearman's coefficient because of non-parametric data with bias index. These correlations indicated that the larger attention allocation bias to the fellow eye, the greater interocular suppression and the worse visual acuity.

**VI Effect of multiple measurements on training outcomes**

To determine whether repeated measurements affected the training outcomes, we plotted the improvements in both suppression and logMAR acuity against the number of measurements. As shown in Fig. S5, while some participants with repeated measures had the largest improvements, others had the least improvements, particularly for those with the largest number of repeated measurements. There was no correlation between repeated measures and improvements in both Supp. Index (left panel, R=0.29, p>0.05) and logMAR (right panel, R=0.09, p>0.05). Also, there was no significant difference between the groups of pre/post measure and repeated measure in both Supp. Index (p>0.05) and logMAR (p>0.05).

**Insert Fig. S5 about here**

**Fig. S5.** Training outcomes as a function of the number of measurements. A, Supp. Index improvement; B, LogMAR acuity improvement. “n.s.” indicates p>0.05 with Mann-Whitney Test for two independent samples. Correlation coefficients and significances were calculated using one-tailed Pearson's R.

**VII Methods**

**1. Participants**

All participants (n=17) were given eye examinations and were refracted under noncycloplegic condition by one of the authors (C.H.), a pediatric ophthalmologist, before perceptual learning. Visual acuity was measured at 6 m with a logMAR chart (Bailey-Lovie) at the best optical correction, and the measurement lasted about 30 seconds. Normal-vision controls had or were best-corrected to visual acuity of 0.00 logMAR (20/20) in each eye. Eligible participants had best-corrected visual acuity in the amblyopic eye (AE) of 0.20 logMAR (20/30) or worse and in the fellow eye (FE) of 0.00 logMAR (20/20). Stereoacuity was evaluated with the Random-Dot Stereo Butterfly card (Stereo Optical, Chicago, IL, USA) at 40 cm with the best optical correction. Amblyopia was defined as anisometropic amblyopia (≥1 D of refractive error interocular difference; referred to as “Aniso”), strabismic amblyopia, or a mix of both. We refer to strabismic or mixed amblyopia as “Strab” in this study. Note that the participant S6 had strabismus due to congenital IV nerve palsy. His stereoacuity and deviation were measured with the head tilted 10° to the left. Individuals with other eye diseases (e.g., cataract, glaucoma, age-related macular degeneration, etc.) or other types of amblyopia (e.g., secondary to congenital cataract) were excluded in this study. All participants were screened for the presence of monocular fixation instability and eccentric fixation using a direct ophthalmoscope. Participants who had eccentric fixation and nystagmus or latent nystagmus (nystagmus that appears when covering one eye) were excluded from the study.

**2. Perceptual Learning**

***Display and dichoptic setting***

The stimuli were presented at a viewing distance of 85 cm on a pair of Sony Trinitron Multiscan G400 CRT monitors with a frame rate of 85 Hz. An adjustable mirror stereoscope was used to combine the left-eye and right-eye views into a single view, in which both the horizontal and vertical deviations in strabismic amblyopes were adjusted by mirrors to align the nonius lines under the best optical correction. All strabismic amblyopes, except for participant S7, had binocular fusion before the training. During the first 2 visit sessions, participant S7 could not align the central nonius lines but had peripheral fusion of the surrounding noise in the stimuli. However, starting from session 3, participant S7 gained central binocular fusion and could align the central nonius lines.

***Stimuli***

We modified a feature counting paradigm that was used in a monocular viewing study in strabismic amblyopes ^9^ for dichoptic presentation, in which the trained eye was presented with an array of vertical Gabor patches that were randomly place (between 1 and 8 Gabors) as “targets”, while the untrained eye was presented with horizontal Gabors patches (between 1 and 8 Gabors) as “distractors”, with a maximum of 9 patches including both vertical and horizontal Gabors. This design was based on attention framework proposed by previous studies ^10-13^, in which selective attention operates through facilitating neural responses to attended stimuli (i.e., targets) and suppressing neural responses to unattended stimuli (i.e., distractors). We expected selective visual attention improvement to visual input from the trained eye that executes the task (searching for “targets” among “distractors”) over repeated practice (perceptual learning). To reduce the length of the blocks, we excluded target set-sizes of 2 Gabors and 7 Gabors. For Participant A1 and S6, we did not include the smallest target set-size of 1 Gabor. The participants were informed that the possible number of targets was between 1 and 8 and they were not aware of missing target set-sizes. The stimuli appeared in the central visual field (5.6° square frame) surrounded by noise in the periphery (21° x 18° in the visual field), followed by a 200 ms noise mask. A 500 ms-valid attentive cue preceded each trial to the trained eye to guide which eye would get the targets. Importantly, we purposefully avoided challenging low-level visual features (e.g., low contrast and high spatial frequency) in the tasks and made the counting elements (Gabors) highly visible at high contrast (≥ 25%) and low spatial frequency (2 cycle /deg) allowing them to be seen by the amblyopic eye with poor visual acuity. We arranged 90% of the trials so that the cue and targets were in the amblyopic eye and 10% of the trails with cue and targets to the fellow eye, with a random order within a block. The surrounding noise was to encourage peripheral fusion. Participants were required to search for the vertical Gabors among the horizontal Gabors and report the total number of vertical Gabors by button press on the keyboard. To increment the level of attention required across perceptual learning sessions, contrasts in the two eyes and stimulus presentation duration were varied, depending on the training strategy used tor the participant (see “Perceptual learning strategy and procedure” in detail). The trials were self-initiated and the participants were requested to respond as accurately as possible with no time limit; no feedback was given. A central fixation point and nonius lines appeared prior to the initiation of all trials, to ensure that the mirror stereoscope remained properly aligned. If the nonius lines were no longer aligned, participants were given the opportunity to re-adjust.

***Contrast matching to equal perceptual visibility in the two eyes (balanced contrast)***

The participants in the balanced-contrast group were required to adjust the contrast in the two eyes for equal perceptual visibilities before starting perceptual learning in each visit session. Two horizontal sinusoidal gratings (3 cpd, 2.5°) were presented in the upper and lower visual field of each eye through a mirror stereoscope. The observer was unaware which eye sees upper or lower gratings and was asked to match the contrast between the upper and the lower gratings. The contrast in the fellow eye was fixed at 25%, and the contrast that perceptually matched the amblyopic eye to the fellow eye was the contrast balance point for the amblyopic eye. This procedure was repeated 3 times, and the average was defined as balanced contrast.

**References**

1. Ooi, T.L., Su, Y.R., Natale, D.M. & He, Z.J. A push-pull treatment for strengthening the 'lazy eye' in amblyopia. *Curr Biol* **23**, R309-310 (2013).

2. Li, J.*, et al.* Dichoptic training enables the adult amblyopic brain to learn. *Curr Biol* **23**, R308-309 (2013).

3. Epelbaum, M., Milleret, C., Buisseret, P. & Dufier, J.L. The sensitive period for strabismic amblyopia in humans. *Ophthalmology* **100**, 323-327 (1993).

4. Mintz-Hittner, H.A. & Fernandez, K.M. Successful amblyopia therapy initiated after age 7 years: compliance cures. *Arch Ophthalmol* **118**, 1535-1541 (2000).

5. Li, R.W., Ngo, C., Nguyen, J. & Levi, D.M. Video-game play induces plasticity in the visual system of adults with amblyopia. *PLoS Biol* **9**, e1001135 (2011).

6. Polat, U., Ma-Naim, T., Belkin, M. & Sagi, D. Improving vision in adult amblyopia by perceptual learning. *Proc Natl Acad Sci U S A* **101**, 6692-6697 (2004).

7. Polat, U., Ma-Naim, T. & Spierer, A. Treatment of children with amblyopia by perceptual learning. *Vision Res* **49**, 2599-2603 (2009).

8. Ding, J. & Levi, D.M. Recovery of stereopsis through perceptual learning in human adults with abnormal binocular vision. *Proc Natl Acad Sci U S A* **108**, E733-741 (2011).

9. Sharma, V., Levi, D.M. & Klein, S.A. Undercounting features and missing features: evidence for a high-level deficit in strabismic amblyopia. *Nat Neurosci* **3**, 496-501 (2000).

10. Desimone, R. & Duncan, J. Neural mechanisms of selective visual attention. *Annu Rev Neurosci* **18**, 193-222 (1995).

11. Geng, J.J., DiQuattro, N.E. & Helm, J. Distractor probability changes the shape of the attentional template. *J Exp Psychol Hum Percept Perform* **43**, 1993-2007 (2017).

12. Adam, K.C.S. & Serences, J.T. History modulates early sensory processing of salient distractors. *J Neurosci* (2021).

13. Geng, J.J. Attentional Mechanisms of Distractor Suppression. *Current Directions in Psychological Science* **23(2)**, 147-153 (2014).
